# Ablations of Ghrelin and Ghrelin Receptor Exhibit Differential Metabolic Phenotypes and Thermogenic Capacity during Aging

**DOI:** 10.1371/journal.pone.0016391

**Published:** 2011-01-26

**Authors:** Xiaojun Ma, Ligen Lin, Guijun Qin, Xinping Lu, Marta Fiorotto, Vishwa D. Dixit, Yuxiang Sun

**Affiliations:** 1 Division of Endocrinology, Department of Internal Medicine, The First Affiliated Hospital of Zhengzhou University, Zhengzhou, China; 2 USDA/ARS Children's Nutrition Research Center, Department of Pediatrics, Baylor College of Medicine, Houston, Texas, United States of America; 3 Digestive Disease Branch, National Institutes of Diabetes, Digestive and Kidney Diseases, National Institutes of Health, Bethesda, Maryland, United States of America; 4 Laboratory of Neuroendocrine-Immunology, Pennington Biomedical Research Center, Louisiana State University System, Baton Rouge, Louisiana, United States of America; 5 Huffington Center on Aging, Department of Molecular and Cellular Biology, Baylor College of Medicine, Houston, Texas, United States of America; Pennington Biomedical Research Center, United States of America

## Abstract

**Background:**

Obesity is a hallmark of aging in many Western societies, and is a precursor to numerous serious age-related diseases. Ghrelin (*Ghrl)*, via its receptor (growth hormone secretagogue receptor, GHS-R), is shown to stimulate GH secretion and appetite. Surprisingly, our previous studies showed that *Ghrl^-/-^* mice have impaired thermoregulatory responses to cold and fasting stresses, while *Ghsr^-/-^* mice are adaptive.

**Methodology/Principal Findings:**

To elucidate the mechanism, we analyzed the complete metabolic profiles of younger (3–4 months) and older (10–12 months) *Ghrl^-/-^* and *Ghsr^-/-^* mice. Food intake and locomotor activity were comparable for both null mice and their wild-type (WT) counterparts, regardless of age. There was also no difference in body composition between younger null mice and their WT counterparts. As the WT mice aged, as expected, the fat/lean ratio increased and energy expenditure (EE) decreased. Remarkably, however, older *Ghsr^-/-^* mice exhibited reduced fat/lean ratio and increased EE when compared to older WT mice, thus retaining a youthful lean and high EE phenotype; in comparison, there was no significant difference with EE in *Ghrl^-/-^* mice. In line with the EE data, the thermogenic regulator, uncoupling protein 1 (UCP1), was significantly up-regulated in brown adipose tissue (BAT) of *Ghsr^-/-^* mice, but not in *Ghrl^-/-^* mice.

**Conclusions:**

Our data therefore suggest that GHS-R ablation activates adaptive thermogenic function(s) in BAT and increases EE, thereby enabling the retention of a lean phenotype. This is the first direct evidence that the ghrelin signaling pathway regulates fat-burning BAT to affect energy balance during aging. This regulation is likely mediated through an as-yet-unidentified new ligand of GHS-R.

## Introduction

Energy homeostasis is determined by the balance between energy intake and energy expenditure; even small differences over prolonged time periods in the balance between energy intake and energy expenditure can result in the development of obesity, insulin resistance and other serious metabolic disorders. Ghrelin is an acylated 28-amino-acid peptide predominantly produced by the X/A-like enteroendocrine cells in the stomach [1], and is the only known circulating orexigenic hormone. Ghrelin increases growth hormone (GH) release, stimulates appetite, and induces fat deposition [2], [3], [4]. Many studies have suggested that ghrelin plays a role in long-term body weight regulation, and changes in circulating ghrelin levels reflect an individual's nutritional status [5], [6], [7]. Ghrelin plays a key role in the regulation of metabolism and energy homeostasis. The biologically relevant receptor of ghrelin is a G-protein-coupled receptor named Growth Hormone Secretagogue Receptor (GHS-R) [8]. By activating GHS-R in the hypothalamus, ghrelin stimulates orexigenic neurons and promotes preferential ingestion of fat [9], [10], [11]. Using GHS-R null mice, we unambiguously demonstrated that GHS-R mediates ghrelin's stimulatory effects on GH-release and appetite [12]. In the last few years, we and others have shown that ghrelin has effects in tissues that do not express GHS-R; thus there are likely other ghrelin subtype receptor(s) apart from GHS-R [13], [14], [15], [16], [17]. We recently reported that *Ghrl^-/-^* mice have impaired abilities to manifest and integrate sleep and thermoregulatory responses under metabolic challenges induced by cold temperature and fasting; intriguingly, *Ghsr^-/-^* mice exhibit the normal responses of their WT counterparts [18]. These observations suggest that, although GHS-R is a biologically relevant ghrelin receptor that regulates GH and food intake, ghrelin and GHS-R also may have independent noncanonical functions, so that the phenotypes of *Ghrl^-/-^* and *Ghsr^-/-^* mice are different.

There are two types of adipose tissues: energy-storing white adipose tissue (WAT) and energy-burning brown adipose tissue (BAT). BAT is primarily responsible for non-shivering thermogenesis in rodents and human neonates; it has emerged recently that variations in BAT activity contribute to differences in energy expenditure even among adult humans [19]. Thermogenesis in BAT is achieved by adrenergic-stimulation of both activation and expression of uncoupling protein 1 (UCP1) in the mitochondria. UCP1, located in the inner mitochondrial membrane, recruits free fatty acid (FFA) into the mitochondria to generate heat [20], [21]. BAT activity is positively correlated with energy expenditure and negatively correlated with body fat content [19], [22]. BAT is responsible for more than half of the body's total oxygen consumption in small animals; these animals contain proportionately greater amounts of BAT when compared to larger mammals, including humans. Increased body temperature of about 1°C could thermodynamically correspond to a 10% increase of metabolic rate [23]. Dysregulation of adaptive thermogenesis in BAT has been shown to impact body fat content, and promote obesity and diabetes [20], [24], [25].

Aging is associated with a loss of lean mass, and is often accompanied by an increase of body fat mass which reflects a positive energy balance leading to weight gain [26]. Furthermore, aging is associated with diminished thermogenic function; aging humans have significantly reduced BAT mass and BAT activity [22], [24], [27], [28], [29], [30]. This blunted thermogenic response also contributes to age-associated obesity and energy imbalance. UCP1 is the key regulator of thermogenesis in BAT. However, genes which activate UCP1 in BAT are not well characterized, and there is hardly any information on the genes that age-dependently regulate thermogenesis.

To further our understanding of the roles of ghrelin and GHS-R in fat metabolism and energy homeostasis, we have analyzed the metabolic profiles and BAT thermogenic capacity of our congenic *Ghrl^-/-^* and *Ghsr^-/-^* mice in detail at both younger and older ages. Our results showed that older *Ghsr^-/-^* mice maintain a youthfully lean phenotype, with a decreased percentage of body fat and increased lean mass; whereas *Ghrl^-/-^* mice do not. Furthermore, this lean phenotype of older *Ghsr^-/-^* mice is independent of difference in food intake or physical activity, but is due to higher energy expenditure. Our data further demonstrated that mitochondrial content and thermogenic regulator UCP1 were elevated in the BAT of the older *Ghsr^-/-^* mice, suggesting increased thermogenesis. To our knowledge, this is the first study which shows that GHS-R has a role in thermogenic regulation of energy-burning BAT in mice. *Ghsr* ablation enhanced thermogenic capacity in older mice, but not younger mice, suggesting GHS-R plays a pivotal role in the aging-related dysfunction of BAT. The differential metabolic phenotypes revealed by *ghrelin*- null mice and *Ghsr*- null mice show the complexity of ghrelin signaling pathway, and suggest the possible existence of additional unidentified GHS-R ligand(s).

## Results

### Reduced body weights and body fat in older *Ghsr^-/-^* mice, but not in older *Ghrl^-/-^* mice

**Figure 1:**
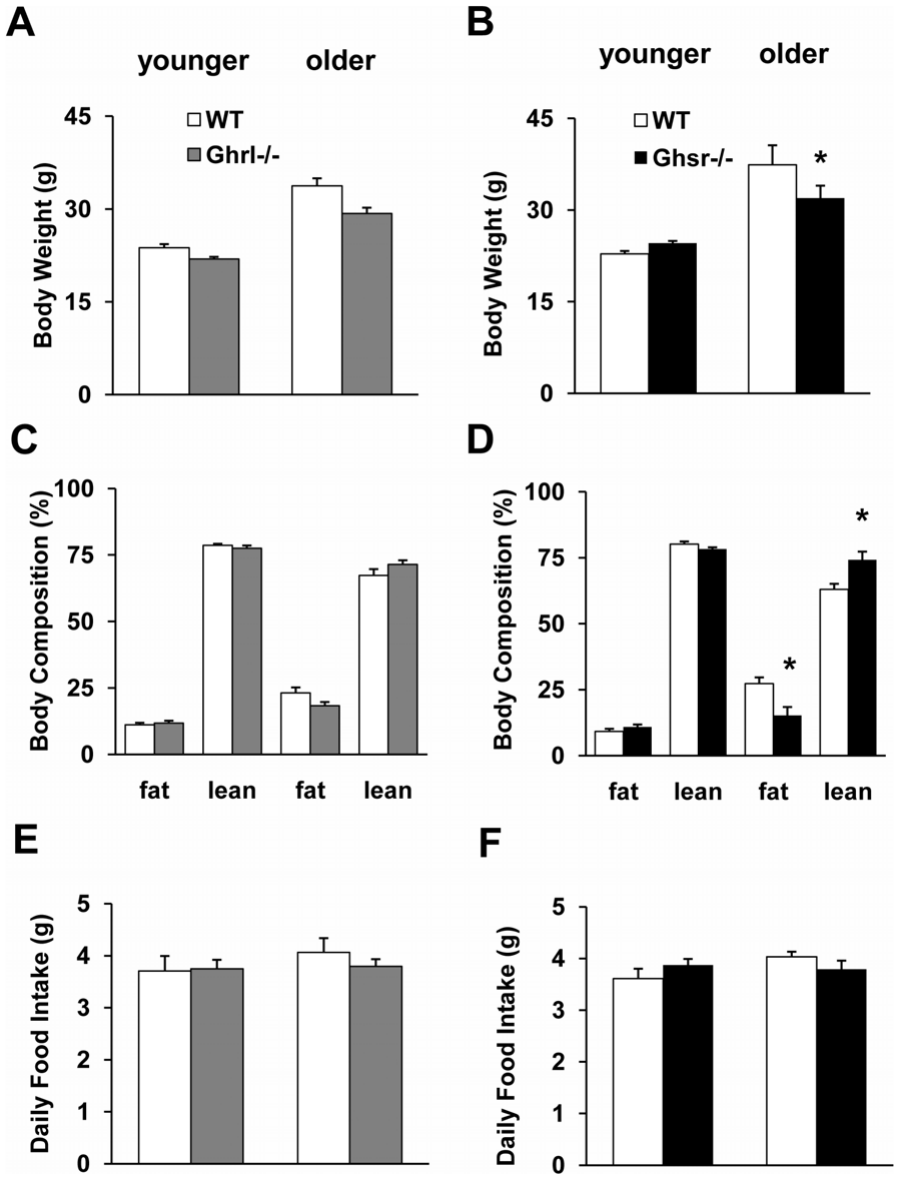
Body weight, body composition and daily food intake of WT, *Ghrl^-/-^*, and *Ghsr^-/-^* mice. The data of Ghrelin-null mice are on the left (A, C, E), and the data for GHS-R-null mice are on the right (B, D, F). (A and B): Both younger and older *Ghrl^-/-^* mice had similar body weight compared to WT mice, while older *Ghsr^-/-^* mice had significantly lower body weight than WT mice. (C and D): The body composition of *Ghrl^-/-^* mice did not differ from age-matched WT mice, while older *Ghsr^-/-^* mice showed decreased proportions of body fat and increased proportions of lean mass. (E and F): The daily food intake of both null mice was comparable to that of their WT controls. The values are presented as mean ± SEM (n = 6–8 per group); *, *P*<0.05, null vs. WT mice.

To characterize the effects on growth and metabolism of deficiency in ghrelin- and ghrelin-receptor (GHS-R), we compared body weights and body compositions of WT, *Ghrl^-/-^* and *Ghsr^-/-^* mice at ages of 3–4 (younger) and 10–12 (older) months. As illustrated in Fig. 1A and 1B, there was no significant difference in body weights between younger null and WT mice, regardless of genotype. We used quantitative nuclear magnetic resonance (NMR) to determine the body composition of the mice. There were no differences among genotypes in body composition at younger age (Fig. 1C and 1D). There were also no significant differences in body composition between older *Ghrl^-/-^* mice and their WT controls (Fig. 1C). In contrast, older *Ghsr^-/-^* mice showed a statistically significant reduction in body weight and fat mass compared with WT controls, while the proportion of lean mass was significantly higher than that of WT mice (Fig. 1B and 1D). Ghrelin is believed to play important regulatory roles in appetite and satiety, and it is logical to assume that *ghrelin* and *Ghsr* ablation will lead to decreased food intake. However, regardless of age there was no difference in the average daily food intake among WT, *Ghrl^-/-^* and *Ghsr^-/-^* mice (Fig. 1E and 1F). These data suggest that the lean phenotype of older *Ghsr^-/-^* mice is not likely due to reduced energy intake (food ingestion).

### Ablation of GHS-R leads to marked increases in energy expenditure and resting metabolic rate in older *Ghsr^-/-^* mice

**Figure 2:**
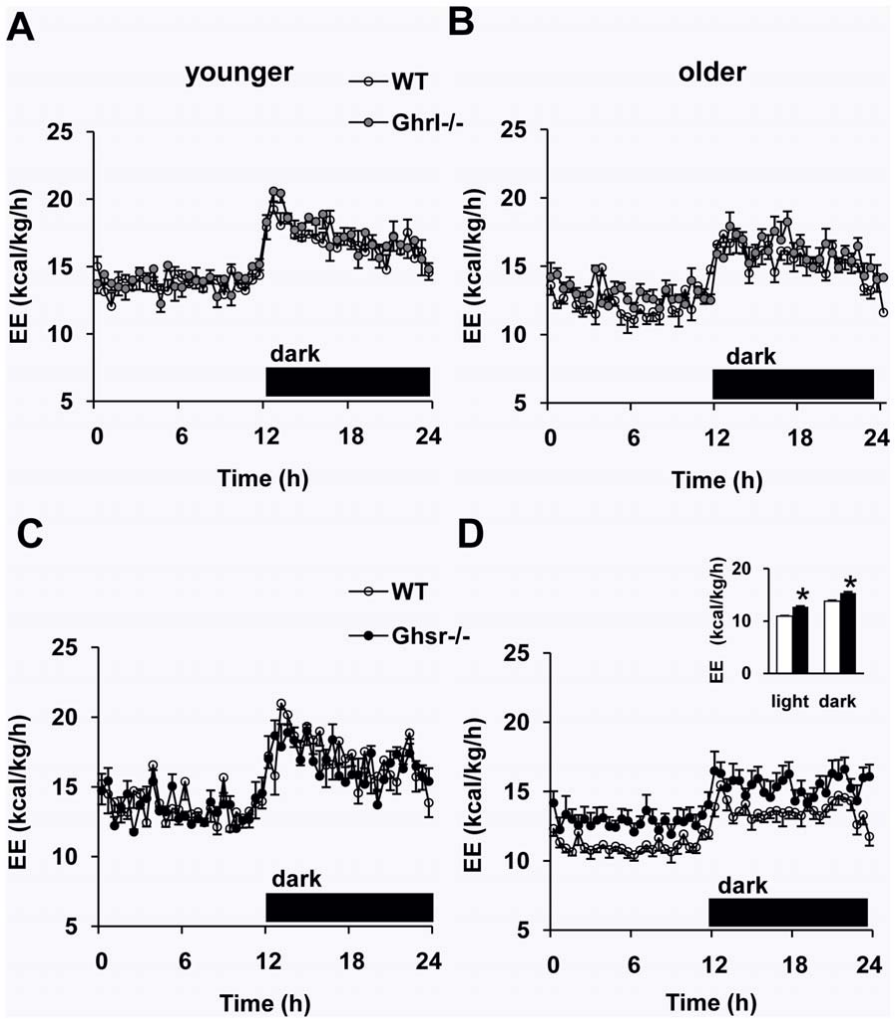
Representative energy expenditure (EE) profiles of WT, *Ghrl^-/-^* and *Ghsr^-/-^* mice. The data for Ghrelin-null mice are on the top (A, B), and the data for GHS-R null mice are on the bottom (C, D). (A and B): The energy expenditure of neither younger nor older *Ghrl^-/-^* mice differed from their WT control mice. (C and D): Older *Ghsr^-/-^* mice had a higher energy expenditure when compared with WT mice, whereas there was no difference in younger mice. The values are presented as mean ± SEM (n = 6–8 per group). *, *P<*0.05, null vs. WT mice.

Changes in body fat mass occur when there is an imbalance between energy intake and energy expenditure. Because food intake was comparable for both *Ghrl^-/-^* and *Ghsr^-/-^* mice compared to their WT counterparts, a potential explanation for the lean phenotype of older *Ghsr^-/-^* mice might be an increase in metabolic rate. We examined the metabolic profiles of younger and older *Ghrl^-/-^* and *Ghsr^-/-^* mice by using an Oxymax/CLAMS system (Columbus Instruments, Columbus, OH). To minimize the confounding effects of stress, mice were caged individually for 1 week and then placed in metabolic cages for at least 4 days before the indirect calorimetry testing. After 24 h of acclimatization in the calorimetry chamber, 48 h of indirect calorimetry data were collected. Both younger *Ghrl^-/-^* and *Ghsr^-/-^* mice had similar energy expenditure levels compared to their WT counterparts (representative 24-hr profiles shown in Fig. 2A and 2C). Older mice in both genotypes showed reduced energy expenditure when compared to younger mice. The energy expenditure of older *Ghsr^-/-^* mice was significantly higher than those of WT mice, and the difference persisted throughout the light and dark phases (Fig. 2D); meanwhile, older *Ghrl^-/-^* mice failed to show a difference (Fig. 2B). We also calculated energy expenditure according to adjusted metabolic body mass (kg^0.75^), which represents the metabolic rate of an animal independent of the size of the body [31], [32]. The adjusted energy expenditure in older *Ghsr^-/-^* mice remained marked higher than that of WT mice (data not shown). Additionally, we determined the respiratory exchange ratio (RER) by indirect calorimetry. There were no differences in RER (Fig. S1), regardless of age and genotypes; this suggests that neither ghrelin-ablation nor GHS-R ablation affects fuel substrate preference under regular chow feeding.

**Figure 3:**
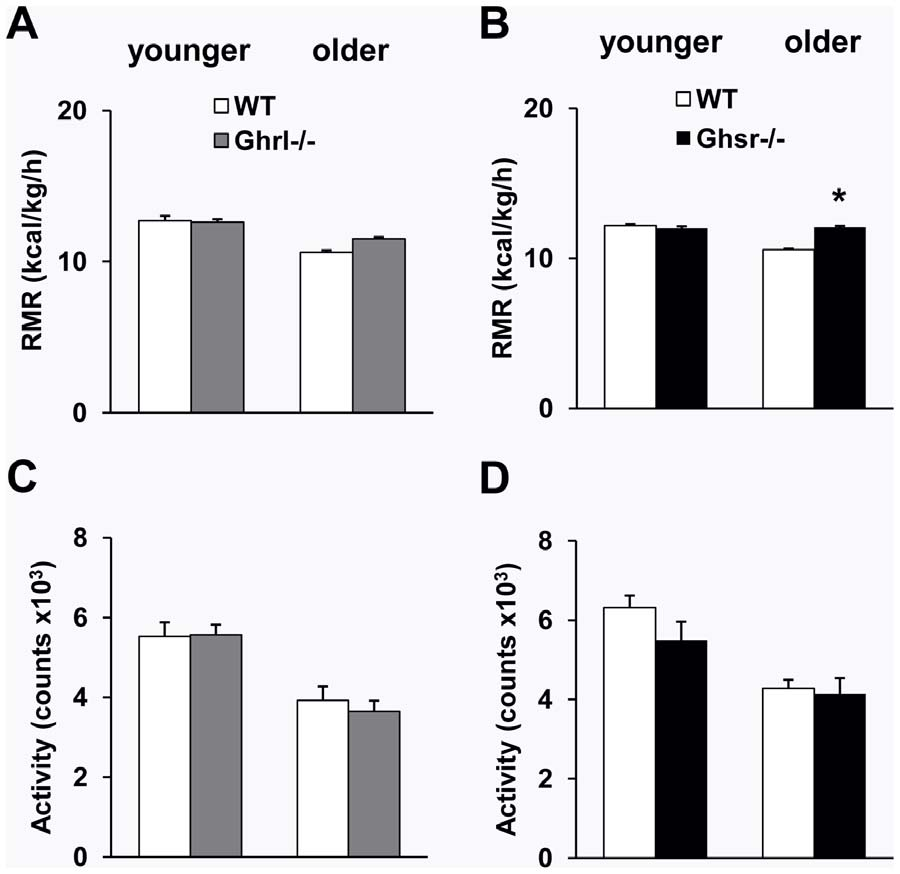
Resting metabolic rate (RMR) and locomotor activity of WT, *Ghrl^-/-^*, and *Ghsr^-/-^* mice. (A): The RMR was not affected in either younger or older *Ghrl^-/-^* mice when compared with WT mice. (B): Older *Ghsr^-/-^* mice had a higher RMR compared with WT mice, whereas younger mice show similar RMR. (C and D): Activity decreases with aging in both mouse models. No significant differences in activity between *Ghrl^-/-^* (C) or *Ghsr^-/-^* (D) mice compared with their WT control mice. The values are presented as mean ± SEM (n = 6–8 per group); *, *P*<0.05 null vs. WT mice.

Resting metabolic rate (RMR) is responsible for burning up to 60–70% of total expended calories [33]. We determined the RMR in these mice to further elucidate whether the differences in total energy expenditure were primarily due to differences in RMR. We found that RMR was comparable between *Ghrl^-/-^* and WT mice, regardless of age (Fig. 3A), but was significantly higher in older *Ghsr^-/-^* mice compared with their WT counterparts (Fig. 3B). These data collectively suggest that the lean phenotype of old *Ghsr^-/-^* mice is primarily contributed by the elevated metabolic rate.

To further determine whether differences in physical activity contributed to the higher energy expenditure/metabolic rate of older *Ghsr^-/-^* mice, we monitored spontaneous locomotor activity of these mice while in the calorimetry chambers. Neither the total daily locomotor activity (Fig. 3C and 3D) nor the locomotor activity during light and dark periods (data not shown) was altered for *Ghrl^-/-^* or *Ghsr^-/-^* mice when compared to their WT controls, regardless of age. Again, the data show that increased energy expenditure of older *Ghsr^-/-^* mice was not due to elevated activity in these null mice.

### Increased mitochondrial content and UCP1 expression in BAT of older *Ghsr^-/-^* mice

**Figure 4:**
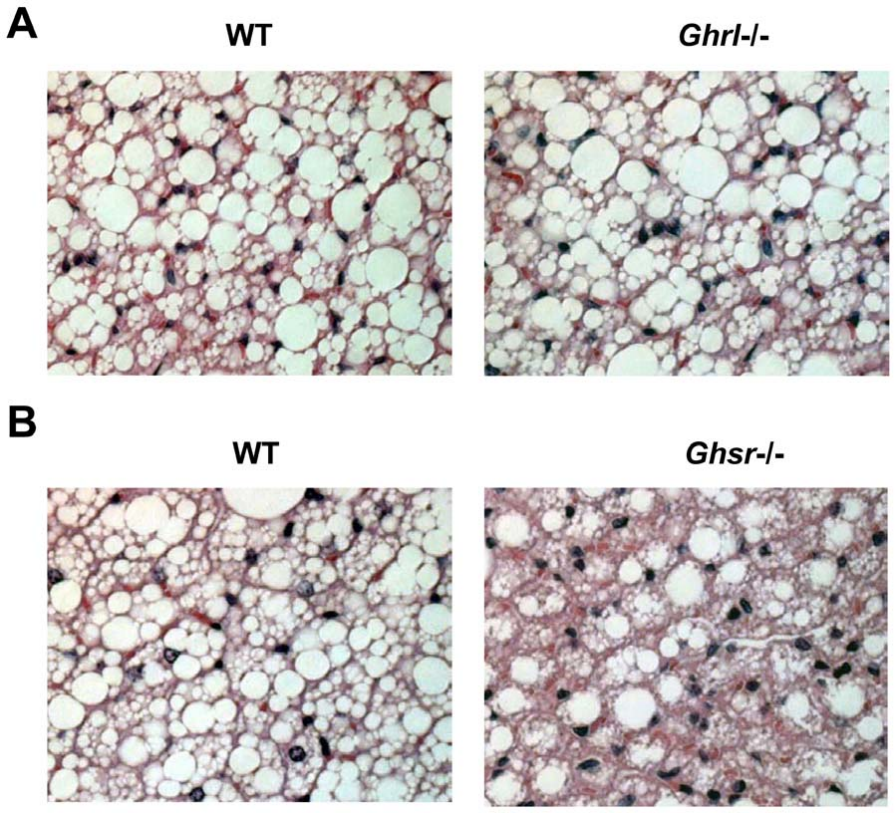
Morphology of interscapular brown adipose tissue (BAT) of older WT, *Ghrl^-/-^*, and *Ghsr^-/-^* mice. (A): The morphology was very similar between older *Ghrl^-/-^* and their WT counterparts. (B): BAT of older *Ghsr^-/-^* mice showed higher percentages of multilobular adipocytes and increased cellularity (dark blue nuclei). These are representative H & E staining of BAT paraffin sections from 4–8 mice.

**Figure 5:**
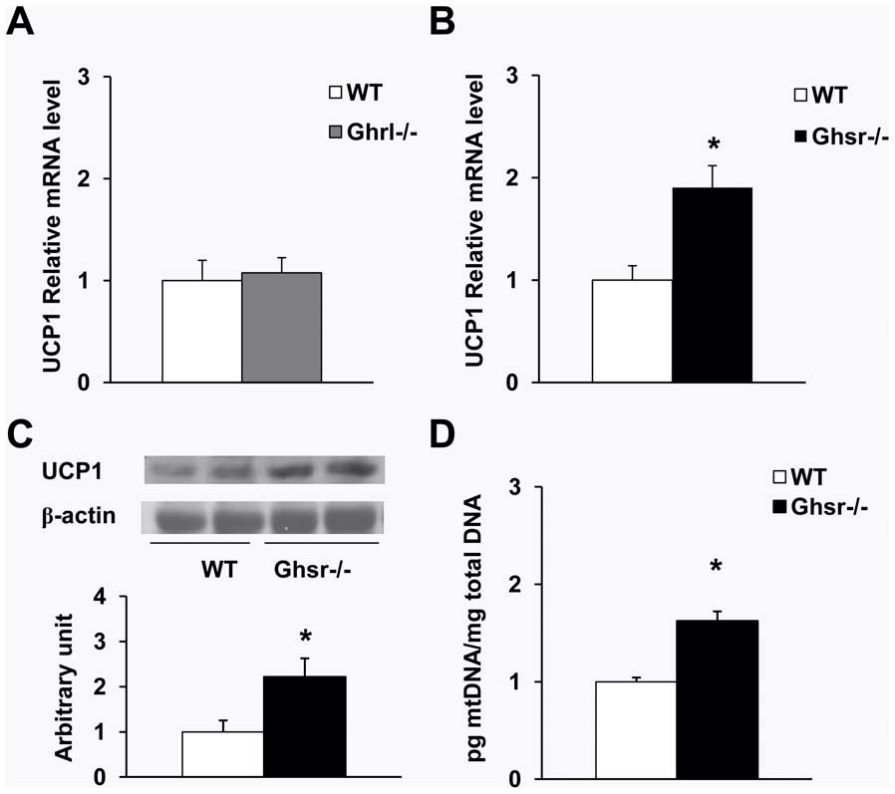
UCP1 expression and mitochondrial DNA content in BAT of older mice. Relative quantitative RT-PCR was used for mRNA analysis, and the expression of average value of WT was defined as 1. (A): UCP1 mRNA expression was not affected in *Ghrl^-/-^* mice. (B): UCP1 mRNA level was increased in BAT of *Ghsr^-/-^*mice compared with WT counterparts. (C): UCP1 protein was increased in the BAT of older *Ghsr^-/-^* mice. Sample Western blots on top and quantization at the bottom. (D) *Ghsr^-/-^* mice had increased mitochondrial DNA content in BAT compared with that of WT mice. The values are presented as mean ± SEM (n = 9–12 per group); *, *P*<0.05 null vs. WT mice.

WAT is composed of large adipocytes that store energy in the form of triglycerides; in contrast, BAT consists of small adipocytes that contain a reduced amount of triglyceride stored in the form of multilobular lipid droplets [20]. The BAT adipocytes have a high mitochondrial density and express the BAT-specific regulator UCP1. Mitochondrial UCP1 is the hallmark regulator of mitochondrial biogenesis and thermogenesis; when activated, UCP1 dissipates the transmembrane proton gradient and generates heat [34]. The H&E staining of BAT sections of older *Ghsr^-/-^* mice showed higher percentages of multilobular adipocytes and increased cellularity (dark blue nuclei) as shown in Fig. 4. To investigate the mechanism of the increased energy expenditure observed in the older *Ghsr^-/-^* mice, we further studied the mRNA expression of UCP1 in BAT of older *Ghsr^-/-^* and *Ghrl^-/-^* mice by quantitative RT-PCR. Consistent with our calorimetry data, no difference was observed in BAT of older *Ghrl^-/-^* mice when compared to that of WT mice (Fig. 5A). Remarkably, however, older *Ghsr^-/-^* mice exhibited significantly increased UCP1 mRNA expression when compared with their WT counterparts (Fig. 5B). Consistently, Western blots showed that UCP1 protein level was increased in older *Ghsr^-/-^* mice (Fig. 5C), further supporting the conclusion of elevated thermogenic function in BAT. We also demonstrated that mitochondrial DNA content was significantly increased in older *Ghsr^-/-^* mice as compared to that of WT mice, indicative of increased mitochondrial content and in line with the enhanced mitochondrial function in the BAT of older *Ghsr^-/-^* mice. These data suggest that GHS-R has a novel role in thermogenesis in BAT, and that GHS-R suppression enhances heat production in BAT. This may in turn lead to increased fat mobilization and results in the lean phenotype observed in older *Ghsr^-/-^* mice.

### GHS-R ablation improves lipid profiles

**Figure 6:**
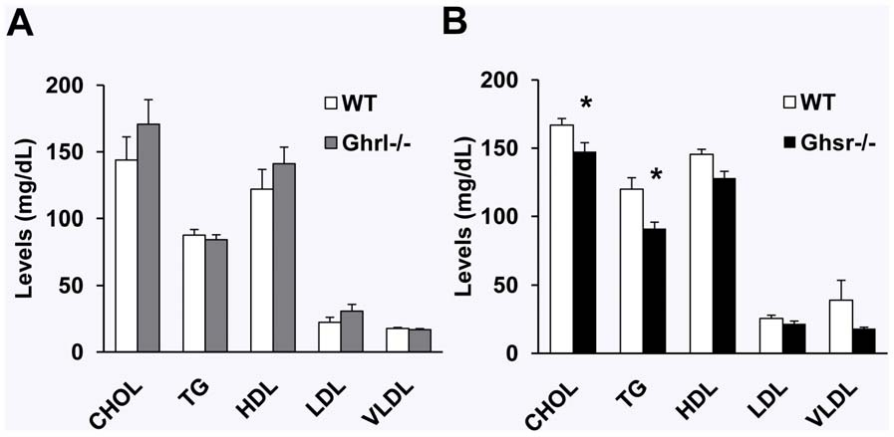
Fasting lipid profile in older WT, *Ghrl^-/-^* and *Ghsr^-/-^* mice. (A): Lipid levels were not different between WT and *Ghrl^-/-^* mice. (B): Levels of total cholesterol (CHOL) and triglycerides (TG) were significantly decreased in *Ghsr^-/-^* mice compared with WT counterparts. The values are presented as mean ± SEM (n = 9–12 per group); *, *P*<0.05 null vs. WT mice.

Obesity is often correlated with dysregulations of lipid metabolism, which ultimately leads to insulin resistance, type 2 diabetes and cardiovascular diseases. The lipid levels were similar between older *Ghrl^-/-^* and WT mice (Fig. 6A). By contrast, in older *Ghsr^-/-^* mice, total cholesterol and triglycerides levels were statistically lower compared with WT mice (Fig. 6B). The data show that ablation of GHS-R improves lipid metabolism. This suggests that old *Ghsr^-/-^* mice may have healthier outcomes.

## Discussion

Pharmacological administration of exogenous ghrelin generates robust orexigenic effects [4], [35], [36]. However, paradoxically, ghrelin levels are lower in obese subjects but higher in lean subjects [37], [38]. The role of endogenous ghrelin in energy homeostasis remains puzzling. We and others have reported that when fed a regular chow diet, younger *Ghrl^-/-^* and *Ghsr^-/-^* mice have normal growth rates, food intake, and body composition, which are not different from WT controls [39], [40], [41], [42]. When the mice are fed a high fat diet right after weaning, *Ghrl^-/-^* and *Ghsr^-/-^* mice appear to prevent diet-induced obesity [41], [42]. Interesting, this protective effect disappears when the high fat diet is fed starting at an adult age [39]. Animals gain weight as they age due to increased fat deposition and reduced energy expenditure [26], [43], [44], [45]. As shown in Fig. 1 of our study, the body weights and proportions of fat mass of the mice increased as the mice aged. Consistent with our previous reports, our present study found no significant difference in body weights among younger (3–4 months old) WT, *Ghrl^-/-^*, and *Ghsr^-/-^* mice. By 10–12 months of age, however, body weights of *Ghsr^-/-^* mice were significantly lower than those of WT mice, whereas body weights of *Ghrl^-/-^* mice were still comparable with same-aged WT mice (Fig. 1A and 1B). During aging, percentage of body fat increases and lean mass decreases. Using NMR, we examined the impact of ghrelin-signaling on body composition during aging. Remarkably, older *Ghsr^-/-^* mice had about 44% less body fat than their WT counterparts, while the difference in older *Ghrl^-/-^* mice was much less pronounced (Fig. 1C and 1D). Our data suggest that GHS-R ablation, but not ghrelin ablation, maintains a youthfully lean phenotype concurrent with aging.

A number of studies have shown that acute administration of ghrelin stimulates food intake, and there are ample results to demonstrate that chronic ghrelin administration resulted in increased fat deposition [4], [36], [46]. However, similar to our previous reports on younger mice [12], [40], daily food intake in older *Ghrl^-/-^* or *Ghsr^-/-^* mice also made no impact on fat deposition (Fig. 1E and 1F). It is clear that the reduction in body weights and changes in body fat/lean ratio in older *Ghsr^-/-^* mice are independent of energy intake (food ingestion).

Imbalance between energy intake (food ingestion) and energy expenditure (activity and/or heat production) leads to changes in body composition [47]. A positive energy balance leads to weight gain, a negative balance results in weight loss, and the weight being primarily composed of fat. It has been shown that ghrelin is involved in the regulation of energy expenditure; further, an inverse relationship exists between ghrelin and energy expenditure in normal-weight humans [48], [49]. Ghrelin induces positive energy balance by stimulating feeding behavior and suppressing sympathetic nerve activity in BAT [50], [51]. Higher levels of ghrelin are associated with lower levels of resting thermogenesis and postprandial thermogenesis in humans [52]. However, it is unknown whether ghrelin's effect on BAT is mediated through GHS-R, and whether *Ghrl^-/-^* or *Ghsr^-/-^* mice have similar BAT phenotypes. As expected, we showed that energy expenditure decreases as the mice age (Fig. 2). Because there was no difference in food intake (Fig 1E and 1F), we speculate that the increased energy expenditure may explain the lean phenotype of older *Ghsr^-/-^* mice. Indeed, older *Ghsr^-/-^* mice exhibited a pronounced elevation in energy expenditure, and also maintained an impressively youthful higher energy expenditure profile when compared with age-matched WT mice (Fig 2D). Because there was no difference in activity level (Fig. 3D), this indicates that in older *Ghsr^-/-^* mice, the increased energy expenditure was primarily due to increased heat production in BAT. In humans, RMR is lower in older individuals than in younger individuals, and that is entirely determined by body composition [53]. Similarly, we detected decreased RMR in older WT mice, while older *Ghsr^-/-^* mice have a significantly higher RMR than that of older WT mice, and to maintain an elevated youthful RMR (Fig. 3B). These data further support the conclusion that the cause of the lean phenotype in older *Ghsr^-/-^* mice is due to increased metabolic rate but not due to changes with appetite or activity.

It has been reported that *Ghrl^-/-^* and *Ghsr^-/-^* mice have decreased RER values with diet-induced obesity, and indicate that the mice preferentially metabolize fat as an energy source [41], [42]. However, our high fat diet study with our congenic mice did not show a difference in RER [39]. In our present study of age-induced obesity, we also did not detect a reduction of RER (Fig. S1). We conclude that ghrelin and GHS-R are not determining factors for fuel preference when regular chow is fed.

BAT plays an important role in energy metabolism, and thermogenic activation of BAT can directly affect metabolic rate. Activation of BAT function is correlated with increased mitochondrial content and activity [54]. We observed that there is no difference in morphology between younger and older *Ghrl^-/-^* mice, whereas the BAT of older *Ghsr^-/-^* mice has an increased abundance of multilobular adipocytes (Fig. 4) and higher mitochondrial DNA content in the BAT of old *Ghsr^-/-^* mice (Fig. 5D). Also, we detected increased cellularity (dark blue nuclei), which suggests that deletion of GHS-R promotes adipogenesis in BAT (Fig. 4). These are all in support of enriched mitochondrial content.

UCP1 is a key regulator of thermogenesis, which allows protons to enter the mitochondrial matrix and dissipates energy as heat in preference to the energy being used for ATP production [20], [21]. Consistent with this histological evidence, we have shown increased UCP1 mRNA and protein expression in the BAT of older *Ghsr^-/-^* mice (Fig. 5B and 5C), but not in older *Ghrl^-/-^* mice. Thus, our data show that *Ghsr^-/-^* mice, but not *Ghrl^-/-^* mice, have enhanced mitochondrial activity. Our data collectively showed increased mitochondrial content and enhanced mitochondrial activity in the BAT of older *Ghsr^-/-^* mice; this may in turn elevate thermogenesis and attenuate the age-associated decline of thermogenic function.

We previously showed that GHS-R expression was not detected in brown adipose tissues of younger mice [55]. Interestingly, our new data show lower level of GHS-R is expressed in BAT of older mice (unpublished data). We have seen increased circulating ghrelin levels during aging [55], which suggest age-related “ghrelin resistance” in older animals. The differential expression patterns in younger versus older mice suggest that GHS-R may be “turned on” specifically in BAT during aging, and may directly regulate the function of BAT. Animal models of fat depot-specific deletion of GHS-R, and/or GHS-R inducible systems turned on during aging, may provide further direct evidence as to whether GHS-R is a key regulator in fat metabolism during aging.

In rodents, it has been shown that BAT may have protective effects during aging. Calorie restriction (CR) is known to have beneficial effects on health and longevity. It has been shown that CR prevents the age-related declines in mitochondrial mass, cyclooxygenase activity and uncoupling levels in BAT of rats [56]. Our data showed that ablation of GHS-R increases thermogenic capacity in BAT, as well as energy expenditure; the increased energy demand created by this enhanced metabolic activity in BAT would support increased fat mobilization, and result in the reduction of body weight. Obesity has many significant deleterious consequences, including lipid dysregulation. We detected an improved lipid profile in older *Ghsr^-/-^* mice (Fig. 6), which further supports the conclusion that older *Ghsr^-/-^* mice have a healthier lean phenotype during aging which placed them at lower risk for diabetes and cardiovascular disease. It is possible that GHS-R is involved in mediating a CR-induced beneficial metabolic state via BAT thermogenesis. Moreover, the inhibition of GHS-R may unravel the beneficial effects of CR on longevity without dieting or excise. To our knowledge, this is the first study using null mice to demonstrate that GHS-R regulates thermogenesis in BAT, thus providing the first evidence that GHS-R regulates BAT thermogenesis, suggesting that GHS-R antagonism may have protective effects against age-induced obesity.

The phenotype we observed in *Ghsr^-/-^* mice could result from GHS-R-mediated effects in central, peripheral and/or hormonal levels. Thyroid hormones are important regulators of RMR and thermogenesis [57], [58]. However, serum T_3_ and T_4_ levels were comparable in WT and *Ghsr^-/-^* mice (unpublished data), suggesting that the elevated RMR of older GHS-R null mice is not due to changes in circulating thyroid hormones; nevertheless, we cannot preclude the possibility of enhanced thyroid hormone activity in *Ghsr^-/-^* mice locally, at the hypothalamic level [59]. GH is another known regulator that plays important roles in adiposity. There is a strong inverse association between visceral fat accumulation and blunted GH secretion in adults [60], [61]. GH is the primary regulator of IGF-1. We previously showed that GHS-R mediates the GH stimulatory effects of ghrelin, so it was expected when older *Ghsr^-/-^* mice showed reduced IGF-1 (Fig. S2). It was surprising, however, that IGF-1 levels in older *Ghrl^-/-^* mice were unchanged when compared to that of WT mice. The reduced IGF-1 level suggests that GH is likely decreased in the older *Ghsr^-/-^* mice, but it cannot explain the lean phenotype. We thus conclude that neither thyroid hormone nor the GH signaling pathway is likely to be mediating the adipose phenotype.

It is intriguing that we have observed differential thermogenic phenotypes in older *Ghsr^-/-^* and *Ghrl^-/-^* mice. Even though GHS-R is considered a physiologically relevant receptor for ghrelin, our current data of differential metabolic phenotypes of older *Ghrl^-/-^* and *Ghsr^-/-^* mice challenges current dogma. Our study adds to the increasing body of literature that there is another as-yet-unidentified GHS-R ligand(s) besides ghrelin that regulate thermogenic function in BAT. Three peptides are derived from the preproghrelin gene: ghrelin (acylated ghrelin), des-acyl ghrelin and obestatin [62]. Acylated ghrelin, which contains an *n*-octanoic acid at the third Ser residue, activates GHS-R. In contrast, des-acyl ghrelin and obestatin do not activate this receptor. Since des-acyl ghrelin does not activate GHS-R, it initially was thought to be biologically inactive. Subsequent studies demonstrated that in addition to acylated ghrelin, des-acyl ghrelin also stimulates lipid accumulation in human visceral adipocytes [63]. Furthermore, both acylated ghrelin and des-acyl ghrelin have adipogenic effects in bone marrow, while a synthetic GHS-R agonist does not; this suggests that acylated ghrelin and/or des-acyl ghrelin activate receptors other than GHS-R [64]. We have shown that des-acyl ghrelin has ghrelin-like effects on feeding which are independent of the activation of GHS-R, and is mediated through orexin [15]. In contrast to ghrelin, obestatin suppresses appetite and enhances energy expenditure [62] and has a leptin-like role in the regulation of metabolism [65], [66], [67]. It was reported that chronic treatment of ghrelin decreases UCP1 expression in BAT [68]; but it is unknown whether the effect is mediated through GHS-R. We recently reported that *Ghrl^-/-^* mice, but not *Ghsr^-/-^* mice, are hypothermic under cold and fasting stress; intriguingly, obestatin attenuates the hypothermic response of *Ghrl^-/-^* mice [18]. Our *Ghrl^-/-^* mice have neither ghrelin nor obestatin. On the other hand, *Ghsr^-/-^* mice have ghrelin and obestatin, but only ghrelin signaling is blocked in these mice. Our *ghrelin-*null has a BAT phenotype different from that of *Ghsr*-null mice, which invites the questions: Whether ghrelin and obestatin have opposite thermogenic effects on BAT? Whether the BAT phenotype of *Ghsr^-/-^* mice can be explained by unopposed effect of obestatin? These observations highlight the complexity of the ghrelin signaling pathway, further investigation is needed.

In conclusion, our study demonstrates differential effects for deficiency of ghrelin- and GHS-R on body composition and energy homeostasis during aging. The decrease of body fat and increase of lean mass in older *Ghsr^-/-^* mice are independent of food intake or activity; they are due to increased energy expenditure might be resulted from enhanced thermogenic capacity in BAT. Our data demonstrate for the first time that GHS-R regulates fat-burning BAT to regulate adiposity and metabolism without affecting energy intake or activity. Thus, GHS-R plays an important role in energy homeostasis during aging, and GHS-R antagonists may be a paradigm-shifting new class of drugs that can prevent age-associated obesity.

## Materials and Methods

### Animals

All procedures used in animal experiments were approved by the Institution of Animal Care and Use Committee at Baylor College of Medicine. The generation of *Ghrl^-/-^* and *Ghsr^-/-^* mice has been previously described [12], [40]. All mice were on a pure C57BL/6J background, and have been backcrossed to C57BL/6J for 13 generations. Mice were maintained under conditions of controlled temperature (∼75°F) and illumination (12-hour light/12-hour dark cycle, 6am to 6pm) with free access to water and regular mouse chow (TD. 2920X, 16% of calories from fat, 60% from carbohydrates, 24% from protein, Harlan Teklad, Madison, WI). Age-matched male *Ghrl^-/-^*, *Ghsr^-/-^* and their WT controls were used in the studies; mice were 3-4 months old (younger group) and 10–12 months old (older group). The amount of food consumed was monitored daily for 1–2 weeks in a Comprehensive Laboratory Animal Monitoring System (Columbus Instruments, Columbus, OH). All procedures used in animal experiments were approved by the Institution of Animal Care and Use Committee at Baylor College of Medicine.

### Body composition and indirect calorimetry

Whole-body composition (fat and lean mass) of *Ghrl^-/-^*, *Ghsr^-/-^* and WT mice was measured by an Echo MRI-100 whole-body composition analyzer (Echo Medical Systems, Houston, TX). Metabolic parameters were obtained by using an Oxymax (Columbus Instruments, Columbus, OH) open-circuit indirect calorimetry system for 72 h. The first 24 h was considered the acclimation phase, and data were analyzed only for the final 48 h. Mice were individually caged in chambers and given free access to regular chow and water for 1-week prior to the tests. Oxygen consumption (VO_2_) (ml/h), carbon dioxide production (VCO_2_) (ml/h), and locomotor activity (beam break counts) were measured. Respiratory exchange ratio (RER) and energy expenditure (EE, or heat generation) were calculated from VO_2_ and VCO_2_ gas exchange data as follows: RER =  VCO_2_/VO_2_ and EE =  (3.815+1.232×RER) × VO_2_ [69]. Energy expenditure was then normalized to body weight. Locomotor activity was measured on x- and z-axes using infrared beams to count the number of beam breaks during the recording period. Resting metabolic rate (RMR) for each mouse was determined by averaging of lowest plateau region of energy expenditure curve corresponding to resting periods, as previously described [70].

### Histological analysis

BAT was fixed overnight in 10% formalin at room temperature, dehydrated and embedded in paraffin. Then tissue blocks were sectioned at 5 µm for H&E staining. The H&E staining was carried out following the standard protocols [71].

### Analysis of gene expression

BAT was snap-frozen in liquid nitrogen and stored at −80°C. Total RNA was extracted from frozen tissue samples using TRIzol Reagent (Invitrogen, Carlsbad, CA). RNA was subsequently treated with DNase (Ambion, Austin, TX) and its integrity was assessed by performing 1.5% agarose gel electrophoresis in the presence of formaldehyde; its concentration was determined by NanoDrop. RNA was reverse-transcribed using Superscript III First Strand Synthesis System (Invitrogen, Carlsbad, CA). Quantitative RT-PCR was performed in triplicate, as previously described [72]. Mouse UCP1 gene-specific primers were obtained from Applied Biosystems (Mm00494070_m1). 18S and β-actin were used as the housekeeping controls. Relative levels of UCP1 mRNA expression were shown as fold expression between null vs. WT mice.

### Western blot analysis

Tissues were lysed in RIPA buffer with complete protease inhibitor cocktail (Roche Inc.). Protein concentration was determined with BCA protein assay kit (Pierce, Rockford, IL). Twenty microgram protein of each sample was separated by SDS-PAGE and electro-transferred to nitrocellulose membrane for immunoblot analyses. The following antibodies were used: anti-UCP1 (Millipore, 1∶1,000), anti-β-actin (Santa Cruz Biotechnology, 1∶1,000), HRP-conjugated anti-mouse (GE Healthcare UK Limited, 1∶10,000), anti-rabbit (GE Healthcare UK Limited, 1∶10,000). The SuperSignal West Pico Chemiluminescent kit (Pierce) was used for the Westerns.

### Extraction and quantification of mitochondrial DNA

Mitochondrial DNA (mtDNA) was extracted and quantified as described with modification [73]. Briefly, interscapular BAT was dissected and homogenized in isolation buffer (300 mM sucrose, 1 mM EDTA, 5 mM MOPS, 5 mM KH_2_PO4, 0.01% BSA, pH 7.4) with a glass homogenizer. The homogenate was first filtered through a layer of gauze. Nuclear and cell debris fraction was isolated by centrifugation at 800 g for 10 min at 4°C. The resulting supernatant was subjected to centrifugation at 8,000 g for 10 min at 4°C. Aliquots of the nuclear and mitochondrial fractions were digested overnight in lysis buffer (10 mM Tris, pH 8.0, 10 mM EDTA, 10 mM NaCl, 0.5% SDS, 100 µg/ml Proteinase K) at 37°C. Nuclear and mitochondrial DNA was extracted using the phenol/chloroform method. Nuclear DNA was quantified by NanoDrop (ND-1000 Thermo Scientific). Mitochondrial DNA was linearized by digestion with Bcl-I for 3 h at 50°C, and then boiled for 5 min. Samples were centrifuged at 7,000 g for 5 min, and the resulting supernatant was used for subsequent PCR amplification. PCR was performed to amplify a 162-nt region of the mitochondrial NADH dehydrogenase. The primer sequences were 5′-TACACGATGAGGCAACCAAA-3′ and 5′-GGTAGGGGGTGTGTGTTGTGAG-3′. The amplified PCR product of mtDNA and total DNA were quantified by spectrometry (NanoDrop), and the ratio of mtDNA/total DNA was calculated.

### Plasma analysis

Plasma samples were obtained by low-speed centrifugation of blood samples from 24 h-fasted mice. The assay kits were supplied by Roche Diagnostics Corporation, (Indianapolis, IN). Cholesterol and triglyceride were determined by enzymatic colorimetric methods; LDL, HDL, and VLDL were determined by homogeneous enzymatic colorimetric methods.

## Supporting Information

Figure S1 **Respiratory exchange ratio (RER) of younger and older WT,** ***Ghrl^-/-^*** **, and** ***Ghsr^-/-^*** **mice.** (A and B): The RER of *Ghrl^-/-^* mice did not differ from WT mice in either younger or older groups. (C and D): Both younger and older *Ghsr^-/-^* mice show similar RER compared with WT mice. The values are presented as mean ± SEM (n = 6–8 per group).
(TIF)

Figure S2 **Plasma IGF-1 levels in older WT,** ***Ghrl*** **^-/-^ and** ***Ghsr*** **^-/-^ mice.** Plasma IGF-1 levels were similar in WT and *Ghrl^-/-^* mice (A), whereas older *Ghsr^-/-^* mice showed significantly decreased IGF-1 levels when compared with their WT controls (B). The values are presented as mean ± SEM (n = 7–13 per group); *, *P*<0.05 null vs. WT mice.
(TIF)
