# Supplementary figures and images for: Ablations of Ghrelin and Ghrelin Receptor Exhibit Differential Metabolic Phenotypes and Thermogenic Capacity during Aging
Source: PLoS One. 2011 Jan 26;6(1):e16391. doi: 10.1371/journal.pone.0016391 (PMC3027652; doi:10.1371/journal.pone.0016391)

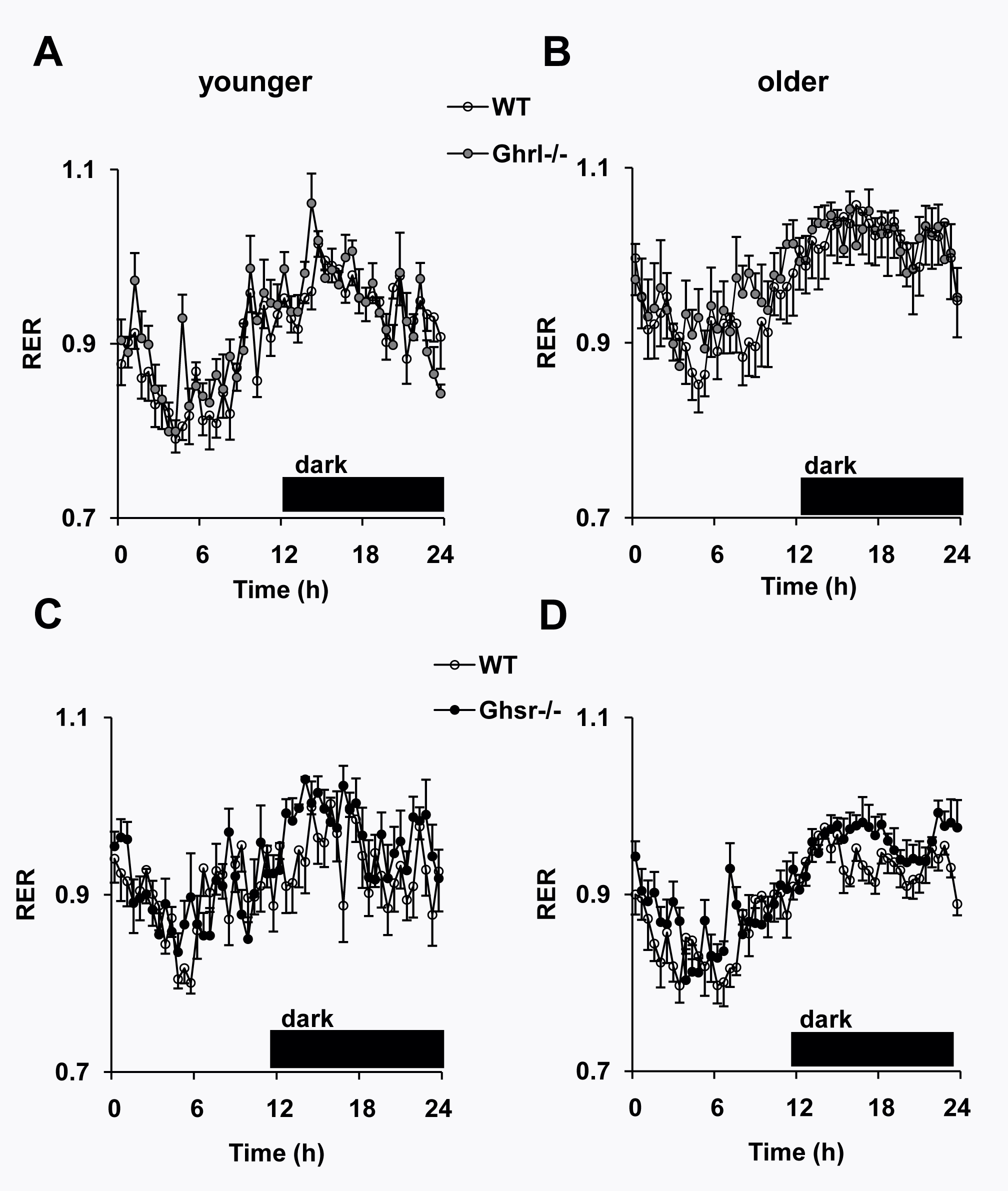

Supplement: Figure S1 — Respiratory exchange ratio (RER) of younger and older WT, Ghrl-/- , and Ghsr-/- mice. (A and B): The RER of Ghrl-/- mice did not differ from WT mice in either younger or older groups. (C and D): Both younger and older Ghsr-/- mice show similar RER compared with WT mice. The values are presented as mean ± SEM (n = 6–8 per group). (TIF) [file pone.0016391.s001.tif]

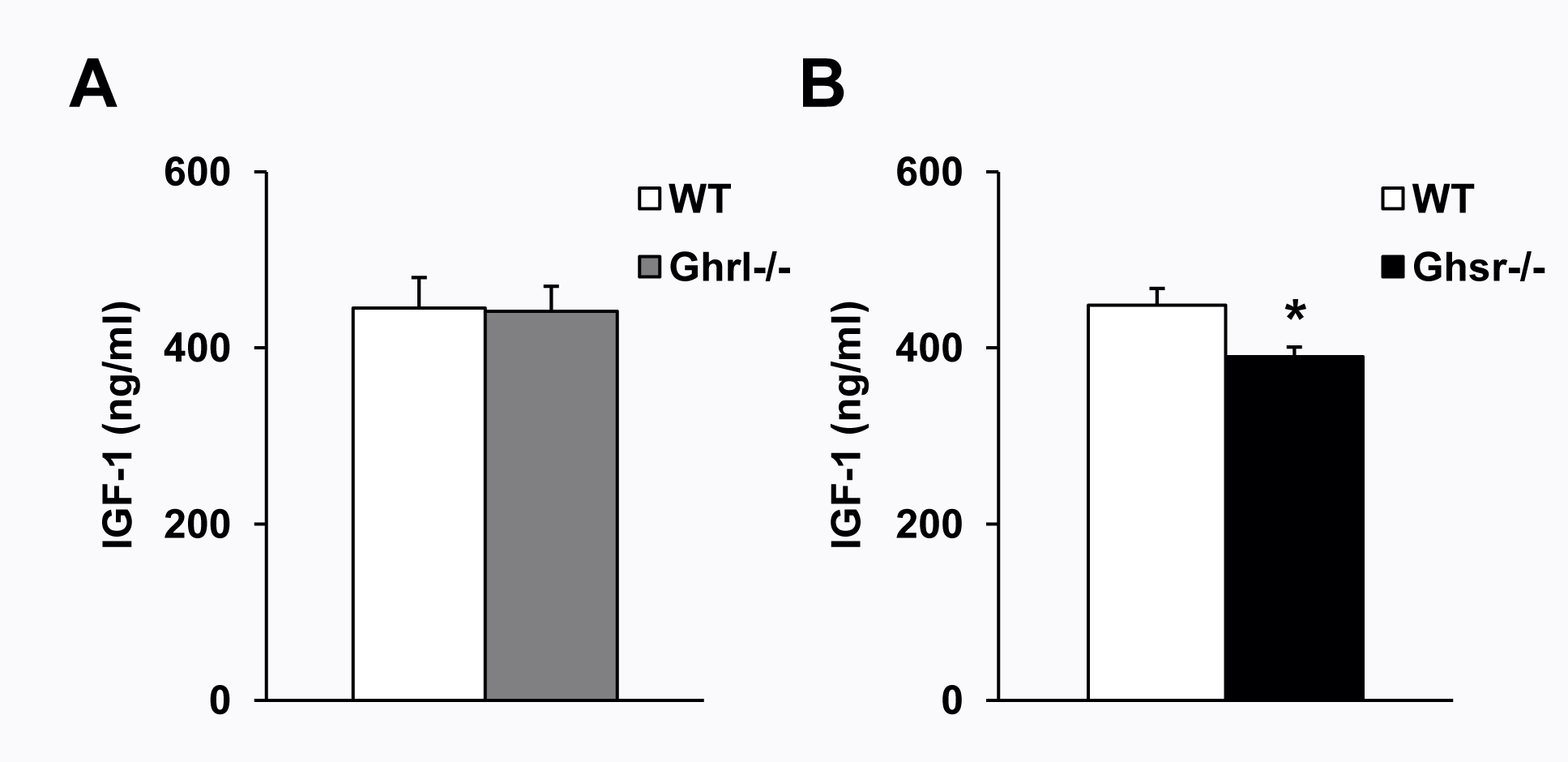

Supplement: Figure S2 — Plasma IGF-1 levels in older WT, Ghrl -/- and Ghsr -/- mice. Plasma IGF-1 levels were similar in WT and Ghrl-/- mice (A), whereas older Ghsr-/- mice showed significantly decreased IGF-1 levels when compared with their WT controls (B). The values are presented as mean ± SEM (n = 7–13 per group); *, P<0.05 null vs. WT mice. (TIF) [file pone.0016391.s002.tif]
